# The silent struggle of ageing with SMI: a narrative review of physical health disparities in older adults with serious mental illness

**DOI:** 10.1007/s00127-025-02963-8

**Published:** 2025-07-25

**Authors:** Jo Howe, Laura Lindsey

**Affiliations:** 1 Pharmacy School, Aston University, Birmingham, UK; 2 School of Pharmacy, Newcastle University, Newcastle Upon Tyne, UK

**Keywords:** Serious mental illness, Physical health, Integrated care, Holistic care

## Abstract

**Purpose:**

Individuals with serious mental illness (SMI) experience a significantly reduced life expectancy compared to peoplewithout SMI, affecting millions worldwide. While cardiovascular disease, diabetes, and metabolic syndrome are well-recognisedphysical health conditions in this population, this article addresses the unique challenges in managing dementia, cancer,menopause, osteoporosis, and oral health in the context of SMI and ageing.

**Methods:**

This article presents a conceptually informed narrative review of ageing-related physical health disparities experiencedby people living with SMI. A narrative review approach was adopted to allow for a targeted and iterative synthesis of evidenceacross five underexplored domains: dementia, cancer, menopause, osteoporosis, and oral health. These domains were selecteddue to their public health significance, under-representation in the literature, and relevance to the lived experiences of older adultswith SMI.

**Results:**

Despite their substantial impact on the well-being of individuals with SMI, these areas often receive less attention.Diagnostic overshadowing, limited specialised knowledge among mental health staff about physical health, and the siloed nature ofhealthcare delivery contribute to delays in diagnosis and treatment.

**Conclusion:**

To improve health outcomes and life expectancy for people with SMI, there is an urgent need for integratedhealthcare approaches. Collaborative models that bridge the gap between mental and physical healthcare are essential to ensuretimely access to holistic care and address the unique needs of this vulnerable population as they age.

## Introduction

Severe Mental Illness (SMI) is a mental, behavioural, or emotional disorder that is diagnosable and causes serious functional impairment which substantially impacts or limits one or more major life activities [1]. People with SMIs such as schizophrenia, non-organic psychosis, personality disorder, bipolar disorder or any other severe and enduring mental illness face significant health disparities including reduced life expectancy and poorer physical health outcomes compared with the general population. On average, they die 15 to 20 years earlier than people without SMI [2]. The mortality rates for people with SMI under 75 are five times higher than for people without [3], and this gap continues to widen over time [4]. Estimates suggest that two in three deaths in people with SMI are from preventable physical illnesses [5]. These disparities are particularly pronounced in ageing-related conditions and are driven by factors such as fragmented care, diagnostic overshadowing and the challenges associated with managing medication.

Cardiovascular disease, respiratory disease, diabetes mellitus and metabolic syndrome are the most commonly reported physical health conditions associated with SMI [6]. However, SMI is associated with an increased risk of developing a number of other conditions related to ageing, which have not received as much attention in the literature. Often, when physical health disparities in people with SMI are assessed, not all aging related conditions are included in the analysis [7]. The risks associated with cardiovascular disease and diabetes are well known, and tools such as the Lester tool are used in practice to reduce the potential risk [8].

Older adults with mental health conditions frequently face barriers in accessing timely and appropriate care [9], these challenges are often intensified for those living with SMI, who may encounter compounded effects of ageing, stigma, cognitive decline, and fragmented service provision when compared against people without SMI [10, 11]. Fragmentation between mental and physical health services, especially in high-income countries, often leads to delayed diagnosis and under-treatment [12]. Collaborative models of care have been suggested as a way to address these intersecting needs, however, a recent Cochrane systematic review concluded that presently insufficient research evidence exists to determine if these models can realise improvements [13]. However, only one of the articles included in the review, a pilot study, attempted to address the physical health needs of patients with SMI, as yet this remains a relatively unexplored area.

This article presents a conceptually informed narrative review of ageing-related physical health disparities experienced by people living with SMI. Rather than undertaking a systematic review of one area, we adopted a targeted and iterative approach to synthesising evidence from five underexplored domains: dementia, cancer, menopause, osteoporosis, and oral health. These domains were selected based on their public health significance, under-representation in existing literature, and relevance to the lived experiences of older adults with SMI.

## Dementia

### Overview

People with SMI have an elevated risk of developing dementia, despite their reduced life span [14–18]. For people aged 66, the prevalence of dementia for those with pre-existing schizophrenia diagnoses is 27.9%, where as for those without a schizophrenia diagnosis the prevalence is markedly lower at 1.3% [19]. Overall, a SMI diagnosis is associated with threefold to fourfold increased risk of dementia [20]. This risk is influenced by multiple factors including genetic predisposition [21], long-term antipsychotic use, and lifestyle factors such as smoking, excessive alcohol use and substance misuse [22]. The development of physical health conditions such as cardiovascular disease, diabetes mellitus and metabolic syndrome and the cognitive impairments associated with SMI [23] also contribute to the heightened risk. Recent research suggests a strong association between pre-existing SMI and later dementia onset, although the causative relationship is still uncertain [18]. Late-onset depression is considered one of the most prominent mental health risk factor for dementia [24]. Furthermore, a diagnosis of SMI within the year preceding dementia onset could either reflect a risk factor or an early manifestation of neurodegenerative changes [16, 18]. Of the SMIs, bipolar disorder increases the risk of developing dementia more than schizophrenia or major depressive disorder [25]. However, the Lancet Dementia Commission notably excluded SMI or psychiatric disorders from its list of dementia risk factors [26]. And conversely a study on the experiences of aging in older adults with schizophrenia excluded people who also had dementia diagnosis [27].

Diagnosing dementia in people with SMI is challenging due to overlapping cognitive symptoms and diagnostic overshadowing, where symptoms are mistakenly attributed to the pre-existing psychiatric condition rather than cognitive decline associated with dementia. This can lead to significant delays in treatment [17, 28] and inadequate support for the person with SMI and their family carers. People with dementia commonly require intensive care, leading to higher rates of hospitalisation, institutionalisation, and emergency department visits, which can be intensified for those with pre-existing SMI due to complex care needs resources [28, 29].

Research on healthcare navigation among people with SMI who go on to develop dementia is limited, though studies indicate that pre-existing psychiatric symptoms elevate dementia related healthcare costs and heighten adverse outcomes [18, 30]. Individuals with SMI are also more likely to experience poor quality care and premature institutionalisation, especially when presenting with challenging behaviours or without robust support systems [28, 31]. People with SMI are less likely to engage with primary care, posing further barriers to dementia care access [28]. Early management of mental health symptoms may reduce dementia risk, though more evidence is required to confirm this [15].

### Considerations for treatment

Many national dementia guidelines [32, 33] lack consideration for individuals with SMI as a pre-existing condition. This oversight may delay early diagnosis and preventative healthcare that is critical in dementia care. Though dementia care teams are multidisciplinary, it is unclear how well these teams are prepared to care for people with SMI, or how mental health practitioners are trained in dementia care and screening. Holistic, integrated treatment approaches, including dementia screening training and awareness of dementia’s prevalence among people with SMI, are urgently needed to promote timely diagnosis and improve outcomes for this vulnerable population and their informal carers.

## Cancer and SMI

### Overview

Cancer is one of the leading causes of death for people with SMI [34]. Cancer prevalence overall in people with SMI is similar to that in those without SMI but mortality is increased 1.4 to 2.0 times for both sexes [35, 36]. The incidence of breast, oesophageal, colorectal, testicular, uterine and cervical cancers are higher in people with SMI whereas they have lower incidence rates of skin, prostate and thyroid cancer than people without SMI [37]. Older women diagnosed with SMI and breast cancer are twice as likely to die from any cause than those without SMI and have a 20% increased risk of death from breast cancer [38]. When comparing people with advanced cancer diagnosis who are hospitalised, people with schizophrenia are dying younger and the time between cancer diagnosis and death is shorter than for people without schizophrenia [39]. This is potentially due to delays in diagnosis of cancer, and people with schizophrenia presenting with more advanced cancer [40]. However, a Danish population level study of cancer incidence and mortality in people with and without SMI concluded the excess mortality of people with SMI was not explained by diagnosis at more advanced stage and treatment effectiveness should be focus of future evaluations. An Australian study found that contact with outpatient and community psychiatry services reduced mortality for cancer and other health conditions [41].

### Considerations for treatment

As a population, people with SMI receive less cancer screening than people without SMI despite the increased mortality [42]. These inequalities in participating in cancer screening increase with age for bowel cancer and cervical cancer [43]. When compared with matched primary care attendees, people with SMI are less knowledgeable about factors increasing cancer risk but more exposed to them [44]. Delayed diagnosis is a potential explanation for the increased mortality [45] as is diagnosis at more advanced stage of cancer and the prevalence of comorbidities [46]. For prostate cancer, patients with pre-existing SMI, are less likely to receive definite initial treatment within a year of diagnosis and have poorer cancer-specific survival after five years of diagnosis than those without SMI diagnoses [47].

The disparity experienced at diagnosis and treatment stages continues to end of life care. Hospitalised cancer patients with SMI are less likely to have their end-of-life concerns documented than cancer patients without SMI even though no significant differences were identified in their decision-making capacities [48]. The end-of-life care received by patients with advanced cancer differs if they also have a schizophrenia diagnosis; in the last month of their life, cancer patients without mental health diagnoses are more likely to receive high-intensity end of life care such as surgery or chemotherapy whereas those with schizophrenia were more likely to receive palliative care [39]. It has been suggested that the reluctance to intervene via chemotherapy or surgery is potentially caused by misconceptions by the health care providers about lack of capacity to consent [49]. Additionally, a qualitative study on palliative care highlighted that people with SMI received inadequate advance care planning [50]. Misguided assumptions and lack of advance care planning are considered to be contributing factors towards the disparity of care at end-of-life [51]. People with SMI who are acutely ill with cancer may also reject treatment or do not fully understand its purpose [49]. They also prefer taking a passive role in decision-making process [52]. For people with SMI, quality of life is priority over life years irrespective of their understanding of cancer and the experiences they have had with health care professionals [53].

A study with palliative care nurses and psychiatric nurses highlighted the need for better knowledge and expertise to improve end-of-life care for people with SMI, with the location for receiving the care being less important than ensuring effective collaboration between the teams [54]. The importance of being proactive and having a team approach across primary and mental health care in the end of life care for people with SMI was also identified in a systematic review [55]. However, currently, we do not fully understand what people with SMI would like at this part of their life stage as there is a lack of representation of the voices of people with SMI and cancer and their significant others in the existing knowledge base [53].

## Menopause

### Overview

Women with SMI experience distinct challenges during menopause that can significantly diminish their quality of life [56]. While symptom improvement is often observed in ageing men with SMI, women frequently experience symptom exacerbation and an increased risk of relapse during menopausal transition [57]. This divergence is largely attributed to the physiological changes, particularly the decline in oestrogen, which shapes the ageing experience of women with SMI [58].

Oestrogen’s neuro-protective effects, implicated in delaying the onset of schizophrenia in adolescence for women [59, 60], decline significantly during menopause potentially destabilising existing SMI symptom management. Women may find their current medications less effective or require higher doses as they transition through menopause [61]. Oestrogen decline also places women at an elevated risk of developing neurodegenerative conditions such as dementia [62] and cardiovascular disease [63], conditions for which people with SMI already possess elevated risk. A study focusing on concerns about menopause of women with SMI found that menopause or approaching menopause made more than half of the participants feel stressed and the five most frequent symptoms experienced by 75% or more of the participants were linked to psychological issues such as feeling depressed, anxious, or worn out, lacking energy or experiencing poor memory [64].

### Considerations for treatment

Hormone Replacement Therapy (HRT), may offer relief for menopausal women with SMI by mitigating oestrogen decline and potentially simplifying SMI treatment regimes [57]. However, diagnostic overshadowing (where menopausal symptoms are misattributed to the SMI) and limited clinician awareness of the menopausal experience in women with SMI creates significant barriers to appropriate treatment [64]. Consequently, clinicians may prioritise mental health stabilisation, potentially overlooking the significant influence of hormonal changes and prolonging recovery. Despite the proven benefits of HRT for general menopausal symptom management, its utilisation in women with SMI remains disproportionately low [57]. Integrated care models encompassing both primary and secondary healthcare services represent a promising strategy for improving access to and quality of care for menopausal women [57, 59]. Such models of service delivery allow for comprehensive assessments of both physical and mental healthcare symptoms which will help to distinguish between the SMI symptoms and menopausal related symptoms, reducing the likelihood of diagnostic overshadowing.

### Osteoporosis: a compounding risk

The elevated risk of physical health conditions in individuals with SMI is further compounded by postmenopausal changes [57]. Osteoporosis, in particular, which results in decreased bone density and an elevated risk of fractures, presents a significant concern, with postmenopausal women with SMI at greater risk of fracture fragility than men with SMI [58, 65]. This vulnerability is multifactorial, encompassing medication side effects, lifestyle factors, and the intrinsic effects of SMI itself. Antipsychotic medications, crucial for managing the SMI, are recognised contributors to bone density loss and fracture risk [66, 67]. A cohort study of individuals aged 50 and over, revealed that SMI diagnosis increased osteoporosis risk by 64% and fragility fracture risk by 87% [68]. Furthermore, the management of osteoporosis in individuals with SMI is complicated by potential medication interactions and the challenges of adherence due to cognitive and behavioural factors.

### Considerations for treatment

Beyond medication, lifestyle factors such as limited exercise, alcohol consumption, smoking and nutritional deficiencies (calcium and vitamin D) contribute to osteoporosis risk [67]. Addressing these factors, requires a multidisciplinary approach, with integrated care models demonstrating efficacy in improving osteoporosis screening and treatment in women with SMI. This highlights the importance of collaborative care across primary and secondary healthcare settings [66, 69]. Again, hampering our understanding of this issue and the potential benefits that may be observed through various treatment options, there is a distinct lack of research exploring service user perspectives of women with SMI who are going through the menopause.

## Oral health

### Overview

Good oral health is part of healthy ageing but it is not prioritised in care development and delivery [70]. Oral health in all ageing adults is often neglected due to comorbidities [71]. However, as a population, people with SMI have a worse oral health than those without SMI, including being nearly three times more likely to have no teeth and having five or more decayed, missing or filled teeth on average than those without SMI [72]. Reasons for reduced oral health in addition to the primary condition include reduction of saliva flow, which is a known side effect of psychotropic medication; lack of awareness of dental problems and prioritising dental care; higher sugar consumption; dental phobia and difficulty in accessing care [73–76]. A qualitative study on oral health experiences of people with schizophrenia found that participants were able to articulate their dental needs; concerns expressed by participants included neglecting oral health when not in a good place, poor dietary habits and the side effects of antipsychotic treatment affecting their dental care such as movement disorder causing their hands to shake and making it challenging to brush their teeth [77].

### Considerations for treatment

As risk factors for poor oral health, such as diet, hygiene, smoking, alcohol use, stress and trauma, overlap with risk factors for other chronic conditions, NHS England and the British Society for Disability and Oral Health have highlighted the importance of the common risk factor approach to improve oral health outcomes for those with SMIs [78]. Following a systematic review, Kisely et al. [79] suggested that an oral health assessment with a standard checklist could be completed by non-dental personnel as a possible intervention for improving oral health. For example, a dental education intervention delivered by dental hygienists for mental health nurses improved both the knowledge of the nurses and the dental hygiene of the patients with SMI [80]. People with SMI have also highlighted practical considerations that would help them to access dental care such as ensuring affordable cost of dental care with no unpredictable costs and collaboration between dentist and mental health services for arranging appointments [81]. To improve dental health, preventative action as early as possible is needed to reduce the long-term impacts. Furthermore, an integrative care model that considers the functional capacity of ageing persons has been suggested as a model for ensuring healthier lives for people who are ageing [82]. Combining all of these approaches would optimise oral health for ageing people with SMI.

## Discussion

This paper highlights five under-recognised domains where ageing individuals with SMI experience pronounced disparities in care: dementia, cancer, menopause, osteoporosis, and oral health. Across these areas, common themes emerge: diagnostic overshadowing, fragmented care systems, and under-prepared health professionals. Although this overview is not systematic, it consolidates existing knowledge and contributes new insights by juxtaposing underexplored health domains rarely reviewed together in relation to SMI.

### Why care for people with SMI is fragmented

Currently, the fragmentation associated with the delivery of health and social care for people with SMI negatively impacts upon their physical health, quality of life and life span [83]. Exactly where the responsibility for the ongoing physical health needs of people living with SMI lies has been heavily debated over the years. The onus is split between services providing mental health, primary care and physical health as well as the individuals, their informal carers and support systems. Each healthcare professional is focused on their area and the overarching perspective to care is missed. More emphasis is needed on ensuring person centred care where the quality and quantity of their lives matter. An example of this would be pharmacists going beyond dispensing medications and using their expertise to ensure patients antipsychotic medication is optimised [84].

### Diagnostic overshadowing

The complexities of managing menopause and osteoporosis in women with SMI, as detailed earlier, exemplify the consequences of fragmented care. Diagnostic overshadowing frequently results in menopausal symptoms being misattributed to mental illness, delaying crucial interventions like HRT. This highlights the necessity for integrated care pathways that ensure holistic care where both mental and physical health needs are addressed. Improving understanding of diagnostic overshadowing may help to improve the suboptimal physical health outcomes for people living with SMI [85]. Invalidation of experiences and not being believed by health care professionals is a concern shared by people with SMI [86]. To effectively reduce diagnostic overshadowing, bilateral improvement of knowledge is needed; mental health workforces need training to screen and understand the complexities of multiple long-term conditions and physical health care providers would benefit from increased awareness of the reality of living with an SMI and the physical effects that antipsychotic medication can have on people.

### Siloed nature of training and delivery of care

Fragmented care is an international problem [87]; the siloed nature of healthcare delivery, with mental and physical healthcare delivered separately exacerbates challenges in diagnosing and delivering holistic care for people with SMI, especially as they age. The disparities in dementia diagnosis and cancer treatment for people with SMI, as discussed, are further compounded by the lack of clear roles and responsibility within the healthcare system. Delays in diagnosis, inadequate palliative care, and poor patient engagement all stem from preventable issues with a system that fails to integrate mental and physical health services. Even when late and non presentation at services has been excluded, professional and service related factors have been found to be a contributing factor to diagnostic errors for their physical health in people with SMIs [88].The already shortened life-expectancy of people with SMI is further reduced by suboptimal care provision.

Fluctuations in mental health stability can create barriers to accessing physical health services, as providers may be hesitant to intervene during periods of acute illness [89].

Although multidisciplinary working has been linked with improvements in care for people with SMI, to truly provide integrated care, staff need to work closely, effectively communicate with one another and have a good understanding of the clarity of roles of the various members of the team [83]. Integrating holistic care into daily clinical practice for people with SMI has been slow [90]. Staff within mental health services lack knowledge, skills and expertise in monitoring and treating physical health [91] which fuels diagnostic overshadowing. Whereas those working in physical health report a lack of understanding of the impact of mental health on the condition [90] which can lead to hesitancy in clinical decision making. Similarly, general practitioners in primary care are not confident in their ability to understand the risk profiles associated with antipsychotic medication [92].

Although mental health nurses strongly believe in the requirement for physical health monitoring to ensure continued good health for their patients living with SMI, and possess a high self-reported confidence in relation to their competency in collating anthropomorphic measurements, such as weight or BMI, and undertaking physical health assessment, such as blood glucose levels, they often report a lack of confidence and a limited understanding of their ability to offer practical solutions for lifestyle management [91]. This lack of confidence and knowledge erodes patient trust and creates a barrier to engagement in essential physical healthcare services. Thus, patients are left living with physical health conditions that are not being appropriately monitored or cared for.

### Stigma associated with SMI

Stigma surrounding mental illness further complicates access to physical healthcare. Carers for people with SMIs have also reported a lack of sensitivity and responsiveness by mental health staff when they have shared about physical health concerns [93]. Conversely, research has also demonstrated that physical health staff possess a lack of understanding about the sequel of SMI and report challenges in communicating leading them to label people with SMI as ‘non-compliant’ [53]. Both healthcare providers and individuals with SMI may hold negative biases that lead to inadequate care [94]. Unmet care needs of older people in general can lead to discrimination and marginalisation [95]. Globally, ageism both at individual and structural levels has been found to affect healthcare provision and reduced health outcomes [96]. However, much of the research into SMI and ageing related conditions is from high income countries. The provision of healthcare for people with SMI living in low- and middle-income countries is even more fragmented and could result in worse outcomes than those already reported in the literature [97, 98]. The combined effect age related discrimination and stigma of SMI will mean older people with SMI are accessing physical health care from a very disadvantaged position.

### Strategies to deliver holistic care

Comprehensive training programs focusing on the interplay between mental and physical health, including the effects of psychotropic medications, are essential to enhance staff competency, build confidence, and mitigate stigmatising attitudes. Care coordinators with physical and mental health training for people with SMI could help to ensure that individuals receive the right care at the right time through organising appointments, facilitating communication between different health and social care services as well as providing education on relevant topics to the individual with SMI. Additionally, more collaborative working between physical and mental health services have been suggested as potentially mitigating factors [99]. Given that people with SMI are likely to experience similar physical health conditions to those without SMI, just at an earlier age [100], then it is imperative that staff in MH settings develop core competencies around physical health parameters [101] to understand what meaningful support with these conditions can be provided within MH services and how and when to make appropriate referrals to other services. Despite increasing awareness of the mortality gap for people with serious mental illness (SMI), there is a dearth of literature exploring the experiences of people with SMI as they age, especially for those who also live with additional physical health conditions such as the ones explored above. Carswell et al. [102], in a UK-based qualitative study, found that individuals living with SMI and co-existing physical conditions often felt that their mental illness compromised their ability to engage with and prioritise long-term physical health management. Participants described focusing on short-term survival rather than future health, with environmental constraints and service fragmentation compounding these difficulties. This highlights an important gap in the literature: there is limited information available to direct and inform treatment regimens and strategies that reflect the lived realities of people with SMI and their informal carers. To reduce health inequalities and improve expectancy of this vulnerable population, more collaborative approaches incorporating the lived experience voice are required to improve service provision and increase the delivery of holistic care. This remains an urgent priority for future inquiry.

### Recommendations for practice and policy

- Integrate physical health monitoring into mental health service protocols, especially for ageing adults.
- Deliver cross-disciplinary training on menopause, dementia, cancer care, and oral health tailored for mental health staff.
- Develop joint care pathways between primary care, specialist physical health services, and mental health services.
- Support family caregivers through targeted education and inclusion in care planning.

## Conclusion

People with SMI are in a disadvantaged position when ageing. Addressing the physical health disparities in older people with SMI requires a fundamental shift towards integrated, collaborative care. By prioritising cross-training, implementing standardised screening protocols, and fostering a culture of shared responsibility, we can ensure that individuals with SMI receive the comprehensive care they deserve. Improving care provision for older people with SMI will improve not only the quality of their life also extend their lifespan as well.

We have drawn on our collective expertise in mental health research, service development, and health inequalities to interpret key themes and offer practice-relevant insights. While the examples and data are drawn primarily from high-income countries. The challenges faced by individuals with SMI in low- and middle-income countries are likely to be greater due to fewer resources and less infrastructure. Future research should explore culturally appropriate interventions and healthcare models suited to resource-limited settings.

## Data Availability

No datasets were generated or analysed during the current study.
